# Homogalacturonans and Hemicelluloses in the External Glands of *Utricularia dichotoma* Traps

**DOI:** 10.3390/ijms252313124

**Published:** 2024-12-06

**Authors:** Bartosz J. Płachno, Małgorzata Kapusta, Marcin Feldo, Piotr Świątek

**Affiliations:** 1 Department of Plant Cytology and Embryology, Institute of Botany, Faculty of Biology, Jagiellonian University in Kraków, 9 Gronostajowa St., 30-387 Cracow, Poland; 2 Bioimaging Laboratory, Faculty of Biology, University of Gdańsk, 59 Wita Stwosza St., 80-308 Gdansk, Poland; malgorzata.kapusta@ug.edu.pl; 3 Department of Vascular Surgery and Angiology, Medical University of Lublin, 16 Staszica St., 20-081 Lublin, Poland; martinf@interia.pl; 4 Institute of Biology, Biotechnology and Environmental Protection, Faculty of Natural Sciences, University of Silesia in Katowice, 9 Bankowa St., 40-007 Katowice, Poland; piotr.swiatek@us.edu.pl

**Keywords:** bladderworts, carnivorous plants, cell wall, cell wall microdomains, Carbotrace 680, external trichomes, hemicelluloses, glands, Lentibulariaceae, scanning transmission electron microscopy, transfer cells, pectic homogalacturonan, xyloglucan, xylan

## Abstract

The *Utricularia* (bladderworts) species are carnivorous plants that prey mainly on invertebrates using traps (bladders) of leaf origin. On the outer surfaces of the trap, there are dome-shaped glands (capitate trichomes). Each such trichome consists of a basal cell, a pedestal cell, and a terminal cell. During the maturation of these external glands, there are changes in the cell wall of the terminal cell of the gland (deposited layers of secondary wall material). Thus, due to changes in the cell wall, these glands are excellent models for studying the specialization of cell walls. The main aim of this study was to check whether different cell wall layers in terminal gland cells have a different composition in the case of homogalacturonans (low-methylesterified HGs, fully de-esterified HGs, and galactan) and hemicelluloses (galactoxyloglucan, xyloglucan, and xylan). The antibodies were used against cell wall components (anti-pectins JIM5, JIM7, LM19, CCRC-M38, and LM5 and anti-hemicelluloses LM25, LM15, CCRC-M1, and CCRC-M138). The localization of the examined compounds was determined using immunohistochemistry techniques, Carbotrace 680, and Calcofluor White. Our study showed the presence of various components in the cell walls of external gland cells: methylesterified and demethylesterified homogalacturonans, galactan, xylan, galactoxyloglucan, and xyloglucan. In the terminal cell, the primary cell wall contains different pectins in contrast to the secondary wall material, which is rich in cellulose and hemicelluloses. We also found that the basal cell differs from the other gland cells by the presence of galactan in the cell wall, which resembles the epidermal cells and parenchyma of traps. A particularly noteworthy part of the cell wall functions as a Casparian strip in the pedestal cell. Here, we found no labeling with Carbotrace 680, possibly due to cell wall modification or cell wall chemical composition variation. We have shown that the apoplastic space formed by the cell walls of the terminal cell is mainly composed of cellulose and hemicelluloses (galactoxyloglucan and xyloglucan). This composition of the cell walls allows the easy uptake of components from the external environment. Our research supports the external glands’ function as hydropotens.

## 1. Introduction

**Figure 1:**
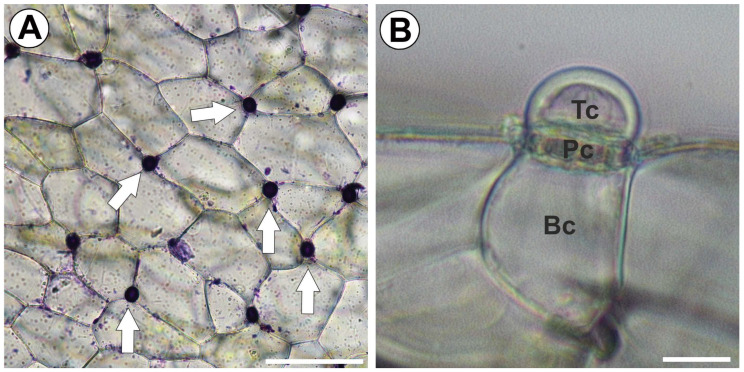
External gland distribution and structure. (**A**) Trap epidermis with external glands (arrows), treated with toluidine blue; the glands absorbed the dye; the bar is 100 µm. (**B**) The structure of the external gland, terminal cell (Tc), pedestal cell (Pc), and basal cell (Bc); the bar is 10 µm.

Plants from the *Utricularia* L. genus are carnivorous plants. Their traps are small elastic–thin-walled vesicles with a mobile trap door that form on various vegetative organs. Both in terms of size and morphology (the structure of the entrance, the presence of various outgrowths, and glands), they are highly variable, which is related to the taxonomic position and the ecological niche occupied [1,2,3,4,5,6]. *Utricularia* traps may also differ in thickness (the number of layers of cells building the trap wall), but, most often, the trap wall consists of an outer epidermis and an inner epidermis [7]. *Utricularia* traps actively capture small animal prey, typically zooplankton (crustaceans, insects, rotifers, nematodes, acari), but also algae and protozoa, to utilize mineral nutrients from prey bodies (e.g., [8,9,10,11,12,13,14,15,16]). The trap works on the principle of fast sucking water together with the animal, which activates the mechanism that opens the door to the trap (by touching the sensory bristles or hairs on the outer surface of the door). This is because, in the trap, there is a negative pressure of ca. −0.12 to −16 kPa relative to the ambient water [17,18,19,20,21,22,23]. Concerning the trap movement duration, *Utricularia* trap movement is considered the fastest among carnivorous plants and one of the fastest in the plant world [21,22,23,24]; for example, Poppinga et al. [25] found that *Utricularia australis* traps captured zooplankton within 9 ms. Westermeier et al. [26] investigated trap biomechanics in 19 *Utricularia* species and found three main trapdoor movement types (with several subtypes). Reifenrath et al. [27] and later also Westermeier et al. [26] suggested that in traps of *Utricularia multifida*, a suction mechanism does not exist and the traps function passively. However, opinions are divided; for example, Lloyd [5] and Płachno et al. [28] believe that the traps of this species are active. It is exciting that in some bladderworts, the traps can open on their own without stimulation by animals [7,29,30,31,32], probably to accumulate organic remains and small organisms that would not be able to open the trap themselves. Water must be removed from the trap lumen to recover the negative pressure so that the trap can function again. After removing about 40% of the water, which takes 25–30 min, the trap is ready to be opened again [18,33]. Sydenham and Findlay [17] and Sasago and Sibaoka [18] showed that water emerges only from the trap entrance region during the resetting. According to these authors, internal glands, mainly bifids, absorb water. However, later, the water must be removed to the external environment. Sydenham and Findlay [17] suggested that either the long-stalked secretory capitate trichomes standing near the free edge of the door or the pavement epithelium were possible structures for removing water. However, Sasago and Sibaoka [18] thought that absorbed water may move across the wall cells of the bladder near the threshold and may be expelled from the capital cells in the outer and middle zones of the pavement epithelium. However, there is another idea about expelling water from *Utricularia* traps. Kruck [34] and Nold [35] proposed that water is expelled by external glands distributed over the trap’s outer surface (Figure 1A,B). This hypothesis has been strengthened by the ultrastructural research of Fineran and Lee [36]. Fineran [37] proposed that the function of external glands changes during trap ontogeny; immature glands take compounds from the environment (acting as root hairs), while mature glands participate in water secretion. Fineran [38] showed that in the terminal cells of these glands, the outer wall is differentiated into several layers, and even cell wall ingrowths are covered by new cell wall layers. Thus, due to changes in the cell wall, these glands are excellent models for studying the specialization of cell walls. Recently, Płachno et al. [39] examined these glands in traps of *Utricularia dichotoma* and found differences in composition between the primary cell wall and the cell secondary wall in terminal gland cells. The outermost layer of the cell wall of the terminal cell, which was cuticularized, was devoid of arabinogalactan proteins (AGPs, recognized by JIM8, JIM14). In contrast, the secondary cell wall in terminal cells was rich in AGPs. However, there is still no information on what other components make up the deposits of the secondary cell wall of these glands. Components that build or impregnate the cell wall affect the transport of aqueous solutions through the cell wall. Hydrophilic components enable such transport, while hydrophobic components inhibit it. That is, the composition of cell walls (outer gland cells) can affect transport both out of the trap and into the trap. Both homogalacturonans and hemicelluloses are hydrophilic components of cell walls. Thus, their presence or absence may have an effect on water expulsion by external glands.

Our main aim for this study is to check whether there are differences in the composition of cell walls within trichome cells in the case of homogalacturonans and hemicelluloses. In this way, we want to supplement the knowledge about the structure of the external glands. It should be noted that a knowledge of structure (ultrastructural features, the presence or absence of cell wall components) helps to understand the function that cells perform within the trichome.

Thus, we hope that our cytochemical studies will help us to understand the role of the external glands.

## 2. Results

### 2.1. Homogalacturonan Distribution

Pectic polysaccharides are found mainly in the middle lamina and the primary cell walls of plant cells. Pectin esterification affects cell wall properties. Therefore, we mainly focused on the detection of pectins with different methylesterification.

**Figure 2:**
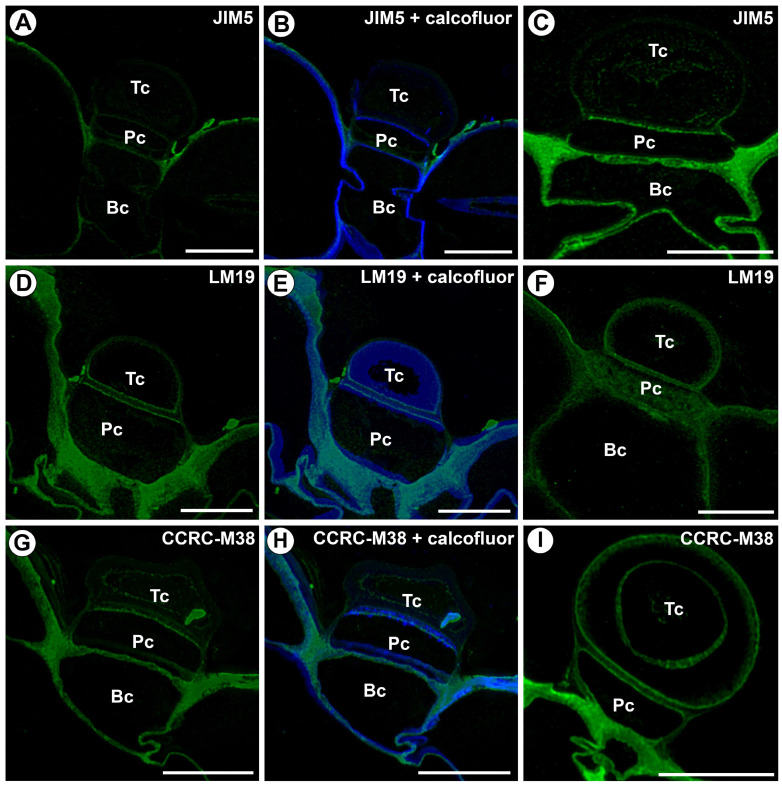
Homogalacturonan distribution in the external gland (intense green color—signal of antibody, blue color—cellulose stained by Calcofluor White), terminal cell (Tc), pedestal cell (Pc), and basal cell (Bc). (**A**) A section through the external gland, labeled with JIM5; the bar is 10 µm. (**B**) The same section as in A, labeled with JIM5 and Calcofluor White; the bar is 10 µm. (**C**) A section through the external gland, labeled with JIM5; the bar is 10 µm. (**D**) A section through the external gland, labeled with LM19; the bar is 10 µm. (**E**) The same section as in (**D**), labeled with LM19 and Calcofluor White; the bar is 10 µm. (**F**) A section through the external gland, labeled with LM19; the bar is 10 µm. (**G**) A section through the external gland, labeled with CCRC-M38; the bar is 10 µm. (**H**) The same section as in (**G**), labeled with CCRC-M38 and Calcofluor White; the bar is 10 µm. (**I**) A section through the external gland, labeled with CCRC-M38; the bar is 10 µm.

The epitope recognized by the JIM5 antibody (low methylesterified HGs) was mainly detected in the cell walls of the basal cell (Figure 2A–C). In terminal cells, this antibody was nearly absent in the outermost cell wall layer, but in the internal cell wall layers, the signal was absent or discontinuous (Figure 2A–C). The fluorescence signal occurred in the transverse walls between the terminal and pedestal cells (Figure 2A–C). There was no fluorescence signal in part of the region of the lateral wall of a pedestal cell (Figure 2C).

The fluorescence signal from LM19 (low-methylesterified HGs) was observed in the terminal cell in the outermost cell wall layer and the external inner cell layer (Figure 2D–F). The fluorescence signal occurred in the transverse wall between the terminal and pedestal cells (Figure 2D–F) and the cell walls of the basal cell (Figure 2D–F).

The fluorescence signal from CCRC-M38 (a fully de-esterified HG) was observed in the terminal cell in the outermost cell wall layer and the innermost cell layer (Figure 2G–I). The fluorescence signal occurred in the transverse wall between the terminal and pedestal cells (Figure 2G–I); however, a more intense signal occurred in the parts of the walls adjacent to the protoplasts. An intense fluorescence signal from CCRC-M38 occurred in the cell walls of the basal cell (Figure 2G–I).

**Figure 3:**
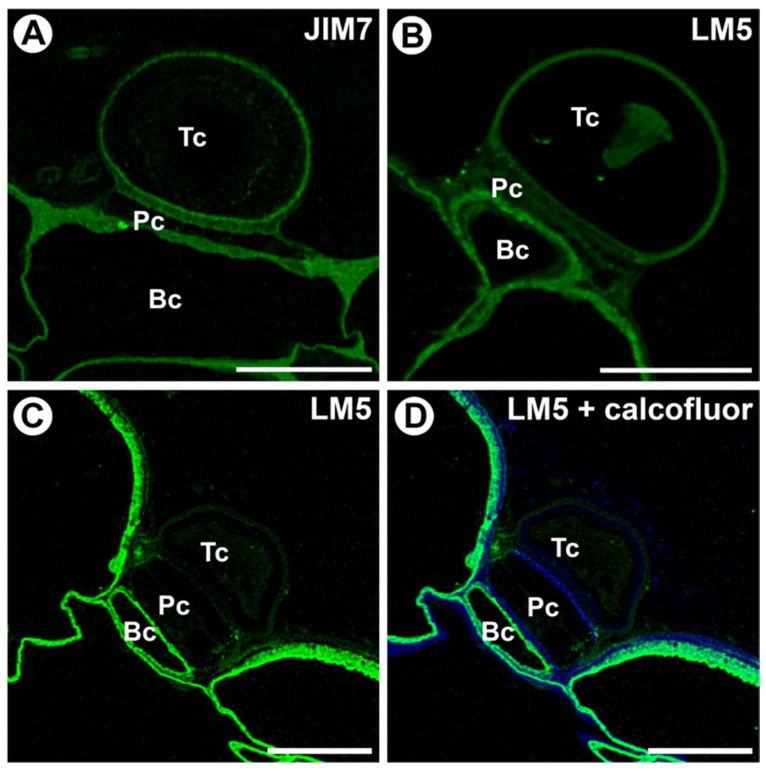
Homogalacturonan distribution in the external gland (intense green color—signal of antibody, blue color—cellulose stained by Calcofluor White), terminal cell (Tc), pedestal cell (Pc), and basal cell (Bc). (**A**) A section through the external gland, labeled with JIM7; the bar is 10 µm. (**B**) A section through the external gland, labeled with LM5; the bar is 10 µm. (**C**) A section through the external gland, labeled with LM5; the bar is 10 µm. (**D**) The same section as in (**C**), labeled with LM5 and Calcofluor White; the bar is 10 µm.

A fluorescence signal from highly esterified HGs (detected by JIM7) was observed in the outermost cell wall layer of the terminal cell (Figure 3A). A delicate signal was present in internal cell wall layers. An intense fluorescence signal was detected in the transverse walls between the terminal and pedestal cells (Figure 3A). A fluorescence signal from these highly esterified HGs occurred in the cell walls of the basal cell (Figure 3A).

The signal from the pectic polysaccharide (1–4)-β-D-galactan (detected by LM5) was observed in the external gland only in the walls of the basal cell (Figure 3B–D).

### 2.2. Hemicellulose Distribution

Hemicelluloses are not only cell wall building materials but also interact with other cell wall components and influence cell wall properties.

**Figure 4:**
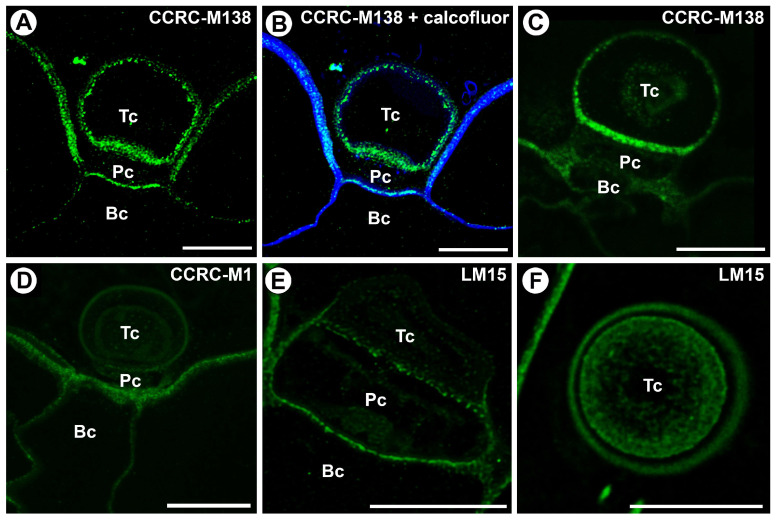
Hemicellulose (xyloglucan) distribution in the external gland (intense green color—signal of antibody, blue color—cellulose stained by Calcofluor White), terminal cell (Tc), pedestal cell (Pc), and basal cell (Bc). (**A**) A section through the external gland, labeled with CCRC-M138; the bar is 10 µm. (**B**) The same section as in A, labeled with CCRC-M138 and Calcofluor White; the bar is 10 µm. (**C**) A section through the external gland, labeled with CCRC-M138; the bar is 10 µm. (**D**) A section through the external gland, labeled with CCRC-M1; the bar is 10 µm. (**E**,**F**) A section through the external gland and through the terminal cell, labeled with LM15; the bar is 10 µm.

The fluorescence signal from CCRC-M138 (which recognizes the glycan group of Xylan-6) was observed in the terminal cell in the outermost cell wall layer (Figure 4A–C). An intense fluorescence signal was detected in the transverse walls between the terminal and pedestal cells (Figure 4A–C).

A fluorescence signal from CCRC-M1 (which recognizes the alpha-Fuc-(1,2)-beta-Gal glycan group of fucosylated xyloglucan) was observed in the cell walls of the basal cell (Figure 4D). The epitope recognized by the LM15 antibody (which reacts with the XXXG motif of land plant xyloglucan) was detected in the cell walls of the basal cell, pedestal cell, and terminal cell (Figure 4E). In pedestal cells, this epitope also occurred in cell wall ingrowths (Figure 4E,F). In the terminal cell, this epitope occurred in the outermost cell wall layer and internal cell wall layers (Figure 2F). However, labeling in the terminal cell depends on the gland and is related to the deposition of the secondary wall (Figure 4E,F).

**Figure 5:**
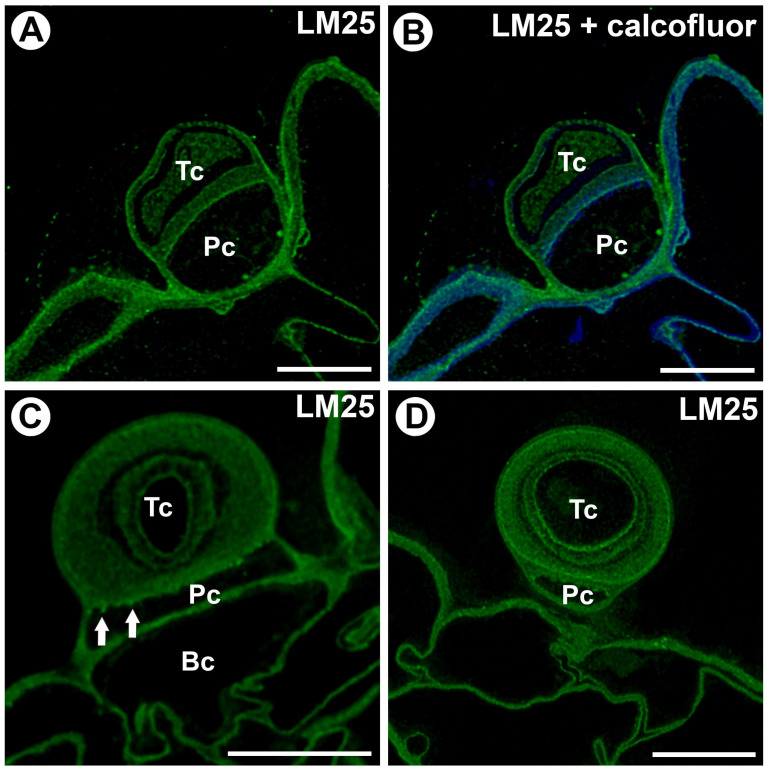
Hemicellulose (galactoxyloglucan) distribution in the external gland (intense green color—signal of antibody, blue color—cellulose stained by Calcofluor White), terminal cell (Tc), pedestal cell (Pc), and basal cell (Bc). (**A**) A section through the external gland, labeled with LM25; the bar is 10 µm. (**B**) The same section as in A, labeled with LM25 and Calcofluor White; the bar is 10 µm. (**C**) A section through the external gland, labeled with LM25, noting the cell wall ingrowths in the pedestal cell (arrow); the bar is 10 µm. (**D**) A section through the external gland, labeled with LM25; the bar is 10 µm.

The epitope recognized by the LM25 antibody (which recognizes galactoxyloglucan) was detected in the cell walls of the basal cell, pedestal cell, and terminal cell (Figure 5A–D). This epitope occurred in the pedestal cell in cell wall ingrowths (Figure 5C). In the terminal cell, this epitope occurred in the outermost cell wall layer and in the internal cell wall layers (Figure 5D).

### 2.3. Histochemistry Staining (Dye Staining)

**Figure 6:**
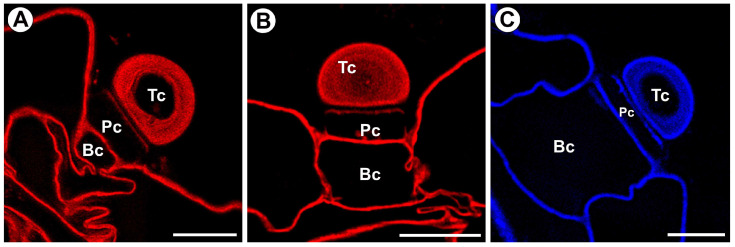
Dye staining the external gland, terminal cell (Tc), pedestal cell (Pc), and basal cell (Bc). (**A**,**B**) A section through the external gland stained by Carbotrace 680 (red color); the bar is 10 µm. (**C**) A section through the external gland stained by Calcofluor White (blue color); the bar is 10 µm.

The cell walls of gland cells were intensively stained with Carbotrace 680, except for cutin-impregnated cell walls (the most external cell layer of the terminal cell, and the region of the lateral wall of a pedestal cell) (Figure 6A,B). The secondary cell wall layers in the terminal cell were intensively stained with Carbotrace 680 (Figure 6A,B).

The cell walls of gland cells were intensively stained with Calcofluor White, except for a cutin-impregnated region of the lateral wall of a pedestal cell and the cutin-impregnated cell wall of the terminal cell (Figure 6C). The secondary cell wall layers in the terminal cell were intensively stained with Calcofluor White (Figure 6C).

## 3. Discussion

Fineran [38], who studied the development of the external glands of *Utricularia monanthos* (=*Utricularia dichotoma* subsp. *monanthos*), showed that the outer wall of the terminal cell usually undergoes extensive secondary wall thickening. He demonstrated that the layers of the secondary wall show a positive reaction for polysaccharide material on staining with periodic acid-thiocarbohydrazide-silver proteinate. Also, Cheema et al. [40] found an accumulation of insoluble polysaccharides in the cell wall of the terminal cell of the external trap gland of *Utricularia stellaris*. Płachno et al. [28] observed that in the external trap glands of *Utricularia westonii* and *Utricularia multifida*, the terminal cell had a thick outer cell wall, which consisted of several layers and contained polysaccharides. This indicates that *Utricularia* species from different sections have a thick cell wall deposition in the terminal cells of the external glands. According to Fineran [37,38], in a fully differentiated trap capturing prey, the thick wall of the terminal cell of external glands forms an apoplastic space, which might provide a suitable environment for maintaining a standing osmotic gradient during water secretion. It is, therefore, of great interest to study how the wall in terminal cells is structured in terms of its constituents. We show here that the outermost part of the cell wall (which constitutes the primary wall) differs from the secondary wall’s layers. These differences relate mainly to the presence of pectin, both de-esterified HGs and highly esterified HGs, because they occur mostly in the outermost cell wall layer. We also found that secondary wall layers are built from hemicelluloses (especially xyloglucan). Interestingly, the occurrence of hemicelluloses in the secondary cell wall coincides with that of arabinogalactans; see Płachno et al. [39]. Arabinogalactans were proposed to act as structural components affecting cellulose deposition through interconnections with other cell wall components, such as hemicelluloses and pectins [41,42]. Hemicelluloses regulate cell wall expansibility and cell-to-cell adhesion [43,44]. An essential biological role of hemicelluloses is their contribution to strengthening the cell wall [45], which may be helpful in *Utricularia* glands, where the protoplast of the terminal cell often degenerates, while the pedestal cell and basal cell are still viable; see Fineran and Lee [36].

According to the manufacturer [46], Carbotrace 680 labels cellulose and hemicellulose xyloglucan. However, some researchers use this tracer to show only cellulose [47,48,49]. In *Utricularia*, staining with Carbotrace 680 was intensive in layers of the secondary cell wall in the terminal cell, which coincides with the detection of hemicelluloses using antibodies. However, Calcofluor White staining showed that the secondary cell wall deposits contain cellulose. Therefore, we posit that the thickened terminal cell wall comprises hemicelluloses (xyloglucan detected by LM15 and galactoxyloglucan detected by LM25) and cellulose. We are excited by the unexpected negative result for cutin-impregnated cell walls from staining with Carbotrace 680. It is still unknown to what extent this part of the wall is different in composition and whether this is due to the availability of cell wall components for the Carbotrace 680. This will require further research into the suitability of Carbotrace 680 for analyzing wall components in walls saturated with hydrophobic substances.

Our work demonstrated that the hemicelluloses recognized by the LM25 (for galactoxyloglucan) and LM15 antibodies (for xyloglucan) were present in cell wall ingrowths in the pedestal cell. This pattern of occurrence of hemicelluloses is also found in cell wall ingrowths in the cells of trichomes and glands of other carnivorous plants: the Drosearaceae species *Aldrovanda vesiculosa* [50,51] and *Dionaea muscipula* [52,53] and the Drosophyllaceae species *Drosophyllum lusitanicum* [54].

According to Fineran and Lee [36,37], in external gland, a local osmotic gradient is created. It allows the transport of water between the pedestal cell and the terminal cell. These authors suggest that one of the structural features that makes it possible to create this gradient is the apoplastic space, which is formed by the cell walls of the terminal cell. In our work, we show that this apoplastic space is composed of cellulose and hemicelluloses (galactoxyloglucan and xyloglucan). On the other hand, this composition of the cell walls allows the easy uptake of components from the external environment, as shown by the use of the vital dye toluidine blue (see also [36]). Thus, external glands function as hydropotens, which are known to occur in many aquatic plants [55].

## 4. Materials and Methods

### 4.1. Plant Materials

*Utricularia dichotoma* subsp. *novae-zelandiae* (Hook. f) R.W.Jobson [56] plants were taken from the collection of the Botanical Garden of Jagiellonian University. The plants were cultivated in wet peat under natural sunlight exposure in a greenhouse.

### 4.2. Histological and Immunochemical Analysis

The traps were cut and fixed, and we later proceeded them according to Plachno et al. [39]. We used the following primary antibodies: anti-homogalacturonans (HGs) JIM5, JIM7, LM19, CCRC-M38, and anti-hemicelluloses LM25, LM15, CCRC-M1, and CCRC-M138 [57,58,59,60,61,62] (purchased from Plant Probes, Leeds, UK). The goat anti-rat secondary or anti-mouse secondary antibody conjugated with FITC was purchased from Abcam (Cambridge, UK). The slides were viewed using a Leica STELLARIS 5 WLL confocal microscope with lightning deconvolution. For JIM5, JIM7, LM5, LM15, LM19, and LM25 staining, a secondary antibody conjugated with FITC was used with an Ex/Em = 495/517 nm wavelength. For CCRC-M1, CCRC-M38, and CCRC-M138 staining, a secondary conjugated with Alexa Fluor 488 was used with an Ex/Em = 493/520 nm wavelength. All photographs were taken with fixed laser parameters, a laser intensity of 14.50% and gain = 28.3 followed by the adjustment of exposure levels set at 12,235/65,535. The counterstain with Fluorescent Brightener 28 (Calcofluor White Stain, Merck Sp. z o.o., Warszawa, Poland) was acquired using an Ex/Em = 358/463 nm wavelength and the fixed laser parameters of a laser intensity of 2% and gain = 2.5.

At least three replications were performed for each of the analyzed traps, and about five to ten sections from each organ were analyzed for each antibody used. Negative controls were created by omitting the primary antibody step (Figure S1). Semi-thin sections were prepared for light microscopy (LM) and stained for general histology using aqueous methylene blue/azure II (MB/AII).

In addition, live traps were sectioned and treated with toluidine blue (Sigma-Aldrich, St. Louis, MO, USA, https://www.sigmaaldrich.com/PL/pl/product/sial/89640 (accessed on 4 December 2024)). The cell wall components, such as cellulose and hemicellulose xyloglucan, were labeled using Carbotrace 680 (Ebba Biotech AB, Nobels väg 16 S-171 65 Solna, Sweden; https://www.ebbabiotech.com/products/carbotrace-680?variant=47885141180748 (accessed on 4 December 2024)). Crystalline cellulose was also labeled using Calcofluor White Stain (Merck Life Science Sp.z.o.o., an affiliate of Merck KGaA, Darmstadt, Germany). Sections were viewed using a Leica DM6000B microscope equipped with a DAPI (Ex/Em = 350/450 nm wavelength; exposure time, 347.136 ms with gain = 1) and Rhodamine filter (Ex/Em = 546/585 nm wavelength; exposure time, 661.156 ms with gain = 1.9).

## 5. Conclusions

Our cytological study showed the presence of various cell wall components in the cell walls of external gland cells: methylesterified and demethylesterified homogalacturonans, galactan, xylan, galactoxyloglucan, and xyloglucan. In the terminal cell, the primary cell wall contains different pectins in contrast to the secondary wall material, which is rich in cellulose and hemicelluloses. We also found that the basal cell differs from the other gland cells by the presence of galactan in the cell wall, which resembles the epidermal cells and parenchyma of the trap. A particularly noteworthy part of the cell wall functions as a Casparian strip in the pedestal cell. Here, we found no labeling with Carbotrace 680, possibly due to cell wall modification or cell wall chemical composition variation.

We show that the thick cell wall of the external gland is a large apoplastic space composed of cellulose and highly hydrophilic hemicelluloses (galactoxyloglucan and xyloglucan). This composition of the cell walls allows the transport of aqueous solutions through the cell wall, i.e., the easy uptake of components from the external environment. Also, this indirectly supports the hypothesis about water expulsion by external glands (as the transport of aqueous solutions can be in both directions). However, further physiological research is needed to confirm this.

## Data Availability

The data presented in this study are available on request from the corresponding author.

## Supplementary Materials

The following supporting information can be downloaded at https://www.mdpi.com/article/10.3390/ijms252313124/s1.

## Abbreviations

AGPs—arabinogalactan proteins, HG—homogalacturonan, LM—light microscope, SEM—scanning electron microscopy, STEM—scanning transmission electron microscopy, XGA—xylogalacturonan.
