# Supplementary material for: Homogalacturonans and Hemicelluloses in the External Glands of Utricularia dichotoma Traps
Source: Int J Mol Sci. 2024 Dec 6;25(23):13124. doi: 10.3390/ijms252313124 (PMC11642213; doi:10.3390/ijms252313124)

Figure S1

Figure S1. Control reactions of cell wall components after immunolabeling (green color – signal of antibody), (**A-B**). Sections through the external glands, stained by Calcofluor White, bar 10 μm.

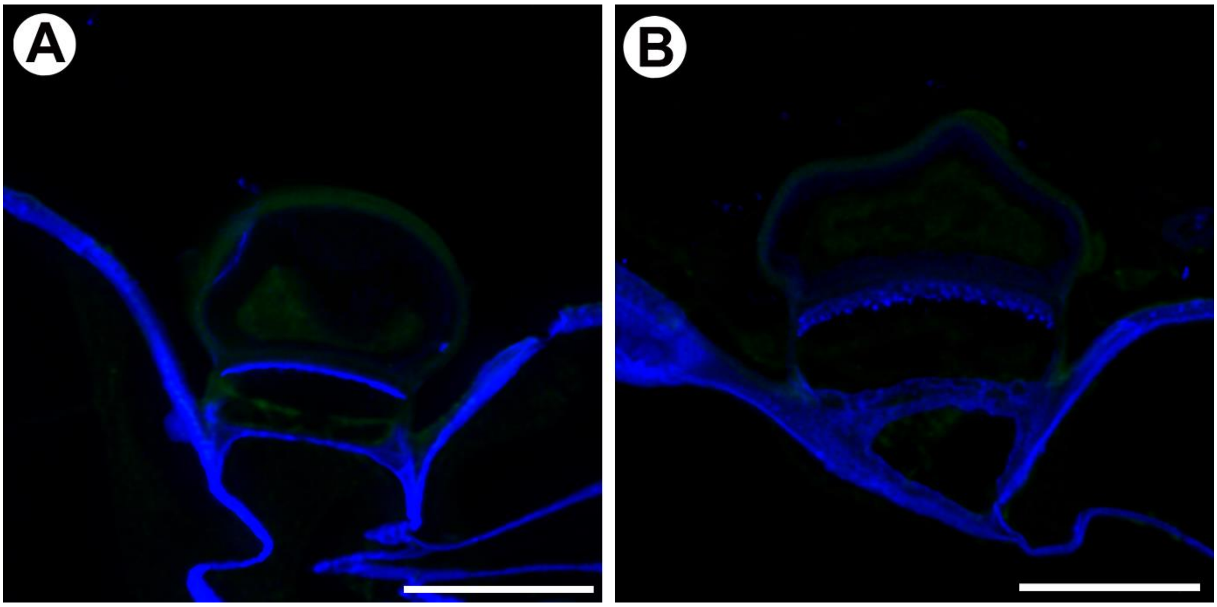

Supplement: Supplementary file 1 [file ijms-25-13124-s001.zip › ijms-3337935-supplementary.pdf]
